# Supplementary material for: A Likelihood Approach for Real-Time Calibration of Stochastic Compartmental Epidemic Models
Source: PLoS Comput Biol. 2017 Jan 17;13(1):e1005257. doi: 10.1371/journal.pcbi.1005257 (PMC5240920; doi:10.1371/journal.pcbi.1005257)
Supplement: S1 File — (TAR.GZ) [file pcbi.1005257.s014.tar.gz › HSPH_Online-SI-Revision/Additions/EnKF08/n100-extreme-SEITR_noisy/n100-extreme-SEITR_noisy_EnKF2_table4.pdf]

1	newly 3 weeks spec	1111.04	4193.04	{42., 17938.}	353.979	0.807168	newly 3 weeks cumul	5404.68	22753.3	{1045., 62562.}	350.841	0.552307	newly 5 weeks spec	368.386	1592.72	{0., 7548.}	426.291	1.22527	newly 5 weeks cumul	6347.06	26962.1	{1145., 81335.}	355.029	0.546072	newly 46 weeks cumul	9732.42	29331.3	{1145., 91482.}	245.28	0.503279
2	newly 3 weeks spec	909.	1380.58	{0., 4576.}	167.083	2.26499	newly 3 weeks cumul	5060.	25364.9	{3123., 85433.}	416.913	0.498045	newly 5 weeks spec	300.	644.08	{0., 1188.}	234.36	2.09046	newly 5 weeks cumul	5879.	26928.	{3225., 85433.}	375.387	0.493642	newly 54 weeks cumul	6291.	28986.3	{3958., 86093.}	378.566	0.515974
3	newly 3 weeks spec	1244.	804.03	{67., 1275.}	76.3256	0.591503	newly 3 weeks cumul	6664.	6887.84	{913., 10738.}	91.0231	0.420668	newly 5 weeks spec	463.	264.4	{1., 415.}	86.3024	0.946364	newly 5 weeks cumul	7872.	7587.35	{917., 11134.}	87.5921	0.431142	newly 56 weeks cumul	8546.	8251.12	{917., 11842.}	87.2637	0.428717
4	newly 3 weeks spec	1162.	1683.22	{0., 3855.}	144.898	1.93787	newly 3 weeks cumul	6474.	18522.9	{2800., 47060.}	215.467	0.366751	newly 5 weeks spec	412.	577.47	{0., 900.}	148.347	1.7714	newly 5 weeks cumul	7613.	20060.8	{2909., 50269.}	193.292	0.35073	newly 54 weeks cumul	8186.	21211.6	{2922., 50703.}	189.049	0.35688
5	newly 3 weeks spec	334.	864.58	{104., 1066.}	190.892	0.343789	newly 3 weeks cumul	2304.	6472.39	{1319., 9415.}	203.908	0.290049	newly 5 weeks spec	78.	199.38	{20., 420.}	200.103	0.537718	newly 5 weeks cumul	2553.	7046.09	{1663., 9703.}	196.496	0.287733	newly 56 weeks cumul	2676.	7541.87	{1760., 10608.}	199.634	0.302009
6	newly 3 weeks spec	210.	2981.07	{214., 12138.}	1330.69	0.731638	newly 3 weeks cumul	1688.	14370.7	{3029., 39521.}	761.454	0.673464	newly 5 weeks spec	40.	1350.04	{14., 5439.}	3300.65	0.903483	newly 5 weeks cumul	1825.	17721.5	{3299., 57756.}	881.098	0.70648	newly 55 weeks cumul	1849.	20770.9	{3391., 80854.}	1033.31	0.744834
7	newly 3 weeks spec	1990.	2206.36	{347., 3951.}	76.7528	0.379981	newly 3 weeks cumul	8888.	21822.7	{7432., 65031.}	157.667	0.302336	newly 5 weeks spec	982.	377.35	{11., 671.}	77.0397	0.936556	newly 5 weeks cumul	11272.	23079.4	{7768., 67402.}	124.565	0.275054	newly 47 weeks cumul	13516.	23415.4	{7808., 67473.}	101.288	0.258291
8	newly 3 weeks spec	1262.	1635.03	{0., 5503.}	129.09	1.97553	newly 3 weeks cumul	6258.	18436.4	{3914., 73149.}	216.25	0.329839	newly 5 weeks spec	510.	641.93	{0., 1219.}	134.739	1.85268	newly 5 weeks cumul	7632.	20065.4	{4534., 77426.}	187.365	0.319467	newly 54 weeks cumul	8594.	21935.2	{4799., 78179.}	183.361	0.345046
9	newly 3 weeks spec	351.	813.96	{52., 1197.}	167.373	0.388843	newly 3 weeks cumul	2373.	4766.31	{2045., 5412.}	113.07	0.291594	newly 5 weeks spec	96.	281.84	{2., 523.}	267.333	0.722712	newly 5 weeks cumul	2626.	5524.76	{2123., 6646.}	123.188	0.299611	newly 56 weeks cumul	2718.	6014.47	{2123., 7726.}	134.519	0.314122
10	newly 3 weeks spec	2417.	1812.11	{256., 4039.}	93.4841	0.623861	newly 3 weeks cumul	10500.	9211.94	{3073., 21167.}	87.5337	0.468107	newly 5 weeks spec	1167.	674.39	{33., 2635.}	94.6298	0.877966	newly 5 weeks cumul	13414.	10983.4	{3444., 29616.}	87.0412	0.50198	newly 52 weeks cumul	16196.	12215.2	{3505., 31892.}	86.9459	0.542558
11	newly 3 weeks spec	2013.	3216.96	{137., 12717.}	148.254	0.585874	newly 3 weeks cumul	9231.	12847.8	{769., 46558.}	122.751	0.509175	newly 5 weeks spec	1003.	1572.37	{17., 2818.}	151.925	0.730118	newly 5 weeks cumul	11702.	16764.6	{951., 69577.}	127.016	0.515667	newly 51 weeks cumul	13920.	19606.5	{1533., 85279.}	122.806	0.49988
12	newly 3 weeks spec	1233.	633.37	{43., 1630.}	75.7737	0.745742	newly 3 weeks cumul	6317.	14916.1	{2108., 56834.}	173.762	0.384025	newly 5 weeks spec	542.	70.43	{0., 205.}	88.8985	1.90067	newly 5 weeks cumul	7681.	15181.5	{2126., 56901.}	140.226	0.361045	newly 52 weeks cumul	8581.	15229.7	{2127., 56902.}	124.946	0.357009
13	newly 3 weeks spec	1573.	1033.68	{21., 2379.}	100.914	0.882955	newly 3 weeks cumul	7548.	9071.59	{1339., 18708.}	102.226	0.416978	newly 5 weeks spec	706.	258.82	{0., 363.}	97.9887	1.59211	newly 5 weeks cumul	9330.	9825.94	{1467., 18730.}	98.3192	0.458953	newly 53 weeks cumul	10733.	10137.9	{1467., 20162.}	95.6551	0.495081
14	newly 3 weeks spec	2628.	1809.73	{26., 7588.}	110.379	0.976638	newly 3 weeks cumul	11099.	24806.5	{5596., 44452.}	142.803	0.340743	newly 5 weeks spec	1313.	705.96	{0., 3193.}	114.143	2.24558	newly 5 weeks cumul	14282.	26595.6	{7069., 54020.}	109.067	0.28227	newly 46 weeks cumul	17443.	27977.9	{8280., 56404.}	90.7773	0.257427
15	newly 3 weeks spec	1421.	1471.98	{392., 2802.}	68.2505	0.349358	newly 3 weeks cumul	6948.	14872.9	{5076., 23894.}	129.147	0.266628	newly 5 weeks spec	621.	296.44	{15., 767.}	71.182	0.763057	newly 5 weeks cumul	8459.	15797.	{5257., 24314.}	110.897	0.265745	newly 47 weeks cumul	9505.	16113.3	{5292., 24314.}	99.7951	0.264638
16	newly 3 weeks spec	1685.	2188.7	{0., 7079.}	177.837	2.78229	newly 3 weeks cumul	8356.	25160.9	{3929., 86202.}	228.64	0.35396	newly 5 weeks spec	644.	262.17	{0., 1227.}	105.346	2.57882	newly 5 weeks cumul	10017.	26215.6	{3929., 86518.}	199.619	0.355964	newly 50 weeks cumul	11020.	26340.6	{3929., 86518.}	184.125	0.364086
17	newly 3 weeks spec	244.	752.15	{222., 1219.}	215.898	0.344778	newly 3 weeks cumul	2213.	4792.22	{917., 6345.}	134.09	0.303102	newly 5 weeks spec	41.	264.45	{37., 531.}	550.463	0.508774	newly 5 weeks cumul	2358.	5490.68	{1270., 7413.}	148.151	0.314443	newly 56 weeks cumul	2381.	6075.39	{1508., 8546.}	167.787	0.331698
18	newly 3 weeks spec	946.	2608.36	{235., 6670.}	213.074	0.381914	newly 3 weeks cumul	4981.	15000.	{2296., 37368.}	225.096	0.397588	newly 5 weeks spec	314.	969.51	{40., 1454.}	281.086	0.574822	newly 5 weeks cumul	5835.	17493.3	{2583., 42020.}	224.019	0.399214	newly 55 weeks cumul	6316.	19832.5	{2683., 43677.}	238.447	0.413895
19	newly 3 weeks spec	2246.	3285.37	{278., 7705.}	116.36	0.405363	newly 3 weeks cumul	9933.	17614.3	{1265., 49740.}	131.356	0.368888	newly 5 weeks spec	1136.	1160.29	{121., 2653.}	89.2967	0.475915	newly 5 weeks cumul	12584.	20708.9	{1576., 55806.}	122.801	0.370351	newly 50 weeks cumul	14784.	23112.5	{1917., 58133.}	113.696	0.359767
20	newly 3 weeks spec	688.	2451.49	{226., 3680.}	298.315	0.393434	newly 3 weeks cumul	3837.	13613.9	{2240., 22249.}	272.541	0.354344	newly 5 weeks spec	219.	687.6	{14., 2381.}	290.374	0.624776	newly 5 weeks cumul	4426.	15562.4	{2421., 34069.}	270.861	0.358311	newly 55 weeks cumul	4693.	17136.8	{2452., 51726.}	285.519	0.374795
21	newly 3 weeks spec	566.	915.28	{213., 1327.}	114.459	0.342858	newly 3 weeks cumul	3401.	4790.42	{1035., 6278.}	82.3916	0.266031	newly 5 weeks spec	156.	426.52	{31., 841.}	227.885	0.517238	newly 5 weeks cumul	3828.	5831.06	{1435., 7111.}	92.6076	0.274092	newly 56 weeks cumul	3988.	7130.66	{1929., 13150.}	115.411	0.290865
22	newly 3 weeks spec	1150.	985.64	{53., 1853.}	87.0104	0.598534	newly 3 weeks cumul	5834.	15739.8	{3088., 38141.}	191.326	0.389312	newly 5 weeks spec	400.	178.26	{1., 263.}	108.43	1.44164	newly 5 weeks cumul	6931.	16319.2	{3093., 45851.}	160.606	0.358566	newly 52 weeks cumul	7573.	16463.	{3093., 45992.}	146.055	0.347871
23	newly 3 weeks spec	361.	2276.35	{303., 4909.}	537.427	0.643127	newly 3 weeks cumul	2348.	14188.1	{7208., 22152.}	505.954	0.694248	newly 5 weeks spec	79.	738.27	{13., 1432.}	863.785	0.849869	newly 5 weeks cumul	2588.	16219.6	{7663., 29997.}	528.428	0.704466	newly 53 weeks cumul	2670.	17212.1	{7684., 31320.}	546.365	0.712104
24	newly 3 weeks spec	617.	2784.9	{327., 8431.}	376.363	0.446326	newly 3 weeks cumul	3939.	15840.6	{2915., 19155.}	318.593	0.402892	newly 5 weeks spec	171.	856.29	{37., 2938.}	441.573	0.53842	newly 5 weeks cumul	4404.	18182.4	{3149., 39617.}	329.934	0.414833	newly 55 weeks cumul	4602.	19902.3	{3194., 73368.}	349.882	0.426748
25	newly 3 weeks spec	1330.	3042.08	{102., 6414.}	219.065	0.805275	newly 3 weeks cumul	7007.	14085.	{913., 55942.}	183.881	0.507824	newly 5 weeks spec	443.	1200.95	{14., 1162.}	259.289	0.843711	newly 5 weeks cumul	8227.	17223.5	{1067., 83317.}	186.604	0.492776	newly 51 weeks cumul	8870.	19541.9	{1141., 86689.}	193.683	0.502854
26	newly 3 weeks spec	920.	443.02	{63., 674.}	84.5978	0.701733	newly 3 weeks cumul	5136.	5956.89	{2389., 5513.}	76.6649	0.249589	newly 5 weeks spec	345.	65.48	{0., 141.}	91.629	1.51725	newly 5 weeks cumul	6057.	6185.7	{2394., 5782.}	77.193	0.295544	newly 49 weeks cumul	6590.	6235.25	{2394., 5837.}	77.4942	0.322941
27	newly 3 weeks spec	2118.	2155.89	{61., 8131.}	124.533	0.735017	newly 3 weeks cumul	9250.	11136.5	{1460., 32352.}	110.028	0.476534	newly 5 weeks spec	1079.	844.18	{3., 1750.}	117.281	1.11043	newly 5 weeks cumul	11803.	13286.5	{1464., 41611.}	110.666	0.513445	newly 49 weeks cumul	14480.	14982.8	{1470., 48395.}	109.729	0.553013
28	newly 3 weeks spec	196.	1193.29	{171., 2258.}	522.536	0.555707	newly 3 weeks cumul	1632.	6917.65	{1019., 18403.}	339.28	0.444956	newly 5 weeks spec	32.	544.86	{28., 792.}	1614.38	0.831713	newly 5 weeks cumul	1750.	8249.06	{1354., 19728.}	383.945	0.46804	newly 56 weeks cumul	1781.	9643.11	{1815., 20037.}	452.339	0.508409
29	newly 3 weeks spec	1912.	1616.3	{108., 3378.}	91.2667	0.594775	newly 3 weeks cumul	9043.	22609.1	{3511., 54767.}	182.414	0.394942	newly 5 weeks spec	849.	232.24	{3., 527.}	92.6572	1.37008	newly 5 weeks cumul	11162.	23429.7	{3554., 57309.}	148.791	0.374374	newly 53 weeks cumul	12786.	23619.7	{3603., 57325.}	128.879	0.364721
30	newly 3 weeks spec	1221.	1061.05	{153., 1881.}	69.6175	0.402132	newly 3 weeks cumul	6812.	7069.04	{1821., 12965.}	59.5831	0.259044	newly 5 weeks spec	428.	421.54	{6., 941.}	98.8271	0.715369	newly 5 weeks cumul	7969.	8139.37	{2132., 14447.}	60.1417	0.264813	newly 56 weeks cumul	8592.	9200.79	{2635., 16342.}	65.3879	0.27401
31	newly 3 weeks spec	1586.	2429.27	{0., 6007.}	134.743	1.28147	newly 3 weeks cumul	7252.	23301.4	{7583., 58444.}	225.594	0.376488	newly 5 weeks spec	689.	718.98	{0., 1845.}	100.267	1.57949	newly 5 weeks cumul	9024.	25323.3	{9531., 58444.}	183.778	0.321442	newly 46 weeks cumul	10290.	26302.9	{10053., 58444.}	157.82	0.283356
32	newly 3 weeks spec	2184.	1137.09	{5., 2185.}	90.4702	1.20514	newly 3 weeks cumul	9583.	18088.5	{4344., 76487.}	122.641	0.281118	newly 5 weeks spec	1094.	310.86	{0., 798.}	95.2121	2.27954	newly 5 weeks cumul	12124.	18950.4	{4565., 76785.}	105.568	0.299516	newly 52 weeks cumul	14626.	19420.3	{4575., 76793.}	94.7467	0.324911
33	newly 3 weeks spec	643.	295.42	{0., 567.}	92.4914	1.57337	newly 3 weeks cumul	3804.	8700.56	{464., 19025.}	162.198	0.410953	newly 5 weeks spec	197.	26.69	{0., 49.}	96.5127	2.27551	newly 5 weeks cumul	4382.	8816.42	{464., 19025.}	137.081	0.384954	newly 56 weeks cumul	4693.	8830.01	{464., 19025.}	128.099	0.384606
34	newly 3 weeks spec	883.	1688.3	{40., 5332.}	163.214	0.586815	newly 3 weeks cumul	4731.	20223.7	{2114., 65352.}	348.314	0.534793	newly 5 weeks spec	278.	263.28	{0., 644.}	103.194	0.977685	newly 5 weeks cumul	5501.	21137.9	{2152., 68417.}	307.36	0.50687	newly 52 weeks cumul	5915.	21330.9	{2155., 68835.}	284.912	0.489621
35	newly 3 weeks spec	239.	941.17	{266., 1887.}	300.121	0.456878	newly 3 weeks cumul	1921.	5504.31	{1115., 9220.}	201.459	0.384077	newly 5 weeks spec	50.	383.72	{44., 797.}	675.56	0.656882	newly 5 weeks cumul	2064.	6473.9	{1523., 13200.}	225.113	0.404079	newly 56 weeks cumul	2096.	7487.26	{2133., 18815.}	264.531	0.436845
36	newly 3 weeks spec	2432.	1431.53	{114., 3055.}	98.1879	0.822175	newly 3 weeks cumul	10891.	7169.41	{633., 15127.}	93.0136	0.673985	newly 5 weeks spec	1211.	621.79	{20., 1734.}	108.052	1.10568	newly 5 weeks cumul	13782.	8734.42	{753., 17951.}	93.6548	0.711448	newly 52 weeks cumul	16479.	9906.15	{806., 27880.}	93.6102	0.749919
37	newly 3 weeks spec	1023.	2571.54	{175., 7372.}	199.011	0.477967	newly 3 weeks cumul	5532.	33526.4	{4737., 113712.}	518.762	0.632857	newly 5 weeks spec	383.	258.98	{6., 507.}	90.9869	0.842868	newly 5 weeks cumul	6497.	34575.4	{4753., 115735.}	446.791	0.592466	newly 53 weeks cumul	7036.	34723.1	{4753., 115848.}	409.412	0.570568
38	newly 3 weeks spec	915.	2207.88	{91., 6960.}	215.53	0.577597	newly 3 weeks cumul	5124.	8116.51	{538., 24013.}	134.209	0.491213	newly 5 weeks spec	323.	1680.33	{32., 5792.}	489.217	0.67595	newly 5 weeks cumul	5962.	11756.5	{669., 34115.}	169.368	0.509227	newly 50 weeks cumul	6391.	18561.5	{866., 73009.}	252.197	0.521576
39	newly 3 weeks spec	1339.	820.32	{133., 1113.}	77.7043	0.508112	newly 3 weeks cumul	6703.	4535.94	{1212., 5643.}	69.8875	0.407254	newly 5 weeks spec	464.	308.12	{27., 346.}	88.194	0.63057	newly 5 weeks cumul	7906.	5341.85	{1336., 6919.}	70.7401	0.416206	newly 52 weeks cumul	8666.	6164.43	{1366., 9550.}	74.924	0.433145
40	newly 3 weeks spec	1822.	2504.12	{243., 7996.}	111.608	0.444217	newly 3 weeks cumul	8114.	22577.7	{2230., 80792.}	206.336	0.378128	newly 5 weeks spec	874.	570.45	{46., 1391.}	90.7952	0.742614	newly 5 weeks cumul	10273.	24303.3	{2238., 86987.}	173.192	0.369163	newly 53 weeks cumul	12291.	24978.4	{2238., 88459.}	152.116	0.384969
41	newly 3 weeks spec	362.	1007.55	{294., 1724.}	189.108	0.391663	newly 3 weeks cumul	2774.	5400.74	{1562., 7134.}	111.006	0.293133	newly 5 weeks spec	74.	424.23	{54., 781.}	483.311	0.612863	newly 5 weeks cumul	2996.	6462.81	{2006., 9770.}	129.843	0.322614	newly 56 weeks cumul	3059.	7530.99	{2085., 12695.}	158.678	0.363881
42	newly 3 weeks spec	2612.	2358.57	{235., 8752.}	90.9322	0.598489	newly 3 weeks cumul	10679.	15327.9	{3330., 38201.}	112.892	0.415259	newly 5 weeks spec	1374.	651.31	{21., 1493.}	90.1157	1.01023	newly 5 weeks cumul	13825.	17130.3	{3431., 45017.}	98.591	0.43134	newly 47 weeks cumul	17141.	17934.9	{3437., 45652.}	87.174	0.456565
43	newly 3 weeks spec	2253.	2207.44	{472., 6257.}	98.2743	0.532414	newly 3 weeks cumul	10165.	10725.9	{2929., 25818.}	86.9108	0.403369	newly 5 weeks spec	1018.	939.82	{87., 3288.}	106.336	0.690764	newly 5 weeks cumul	12779.	13097.4	{3523., 38527.}	89.8407	0.436136	newly 52 weeks cumul	15131.	14900.4	{3793., 45593.}	91.8276	0.471338
44	newly 3 weeks spec	1384.	836.54	{37., 2187.}	97.9263	0.916418	newly 3 weeks cumul	6755.	14399.3	{1768., 39296.}	166.389	0.388761	newly 5 weeks spec	626.	69.79	{0., 181.}	91.4457	1.95556	newly 5 weeks cumul	8295.	14696.8	{1773., 40738.}	142.35	0.40874	newly 52 weeks cumul	9359.	14736.1	{1773., 40922.}	130.864	0.429274
45	newly 3 weeks spec	1196.	1261.53	{0., 1996.}	126.268	1.69591	newly 3 weeks cumul	6409.	18336.6	{2253., 58858.}	223.034	0.406997	newly 5 weeks spec	536.	755.3	{0., 1263.}	163.784	1.96827	newly 5 weeks cumul	7757.	20049.5	{2919., 59268.}	193.499	0.372309	newly 54 weeks cumul	8640.	23135.3	{3089., 78896.}	198.585	0.364599
46	newly 3 weeks spec	1149.	940.62	{267., 1628.}	70.5483	0.43874	newly 3 weeks cumul	6165.	7689.37	{1790., 12979.}	87.9226	0.305323	newly 5 weeks spec	377.	297.15	{15., 750.}	79.7215	0.666561	newly 5 weeks cumul	7203.	8494.07	{2417., 13154.}	81.8174	0.304497	newly 56 weeks cumul	7768.	9468.22	{3155., 15455.}	84.6432	0.311949
47	newly 3 weeks spec	1358.	892.05	{42., 1847.}	91.4455	0.796354	newly 3 weeks cumul	7212.	4045.78	{353., 10232.}	80.6556	0.680292	newly 5 weeks spec	550.	604.93	{5., 1562.}	139.751	1.04212	newly 5 weeks cumul	8640.	5347.87	{354., 11866.}	82.8703	0.690203	newly 51 weeks cumul	9536.	7853.75	{354., 20762.}	100.66	0.722257
48	newly 3 weeks spec	874.	4235.52	{656., 14395.}	397.33	0.482392	newly 3 weeks cumul	5250.	17030.9	{3597., 49497.}	236.574	0.387809	newly 5 weeks spec	248.	2024.95	{132., 7624.}	733.77	0.644366	newly 5 weeks cumul	5990.	22071.9	{4449., 69150.}	279.397	0.418479	newly 54 weeks cumul	6323.	25503.3	{5388., 87306.}	312.665	0.443515
49	newly 3 weeks spec	879.	1093.74	{0., 1891.}	126.046	1.23514	newly 3 weeks cumul	4780.	9322.52	{1536., 18159.}	150.429	0.356426	newly 5 weeks spec	321.	348.83	{0., 997.}	119.654	1.19275	newly 5 weeks cumul	5641.	10283.6	{1829., 22392.}	140.754	0.358244	newly 52 weeks cumul	6148.	10797.7	{1976., 23936.}	135.973	0.36164
50	newly 3 weeks spec	1513.	1759.73	{306., 4447.}	79.6662	0.337346	newly 3 weeks cumul	7746.	14430.	{6072., 29916.}	102.233	0.20163	newly 5 weeks spec	636.	419.1	{31., 1121.}	75.2296	0.646936	newly 5 weeks cumul	9438.	15680.6	{6488., 36405.}	91.4681	0.207841	newly 53 weeks cumul	10556.	16189.1	{6595., 36976.}	87.697	0.225073
